# Epidemiology of eczema in South‐Eastern Australia

**DOI:** 10.1111/ajd.13966

**Published:** 2022-12-19

**Authors:** Berihun M. Zeleke, Adrian J. Lowe, Shyamali C. Dharmage, Diego J. Lopez, Jennifer J. Koplin, Rachel L. Peters, Victoria X. Soriano, Mimi L. K. Tang, E. Haydn Walters, George A. Varigos, Caroline J. Lodge, Jennifer L. Perret, Michael J. Abramson

**Affiliations:** ^1^ School of Public Health and Preventive Medicine, Monash University Melbourne Victoria Australia; ^2^ Allergy and Lung Health Unit, Centre for Epidemiology and Biostatistics Melbourne School of Population and Global Health, University of Melbourne Carlton Victoria Australia; ^3^ Murdoch Children's Research Institute, Royal Children's Hospital Parkville Victoria Australia; ^4^ Department of Paediatrics University of Melbourne Parkville Victoria Australia; ^5^ School of Medicine, University of Tasmania Hobart Australia; ^6^ Department of Dermatology The Royal Melbourne Hospital Melbourne, Parkville Victoria Australia

**Keywords:** atopic dermatitis, cohort studies, eczema, incidence, prevalence, severe eczema

## Abstract

**Background/Objectives:**

Eczema is a common chronic debilitating skin condition in childhood. Data on the epidemiology and natural history of eczema across the life course are lacking. This analysis aimed to describe these epidemiological features in Australian children and adults.

**Methods:**

Data collected on eczema from four Australian cohort studies were analysed: namely HealthNuts, Melbourne Atopic Cohort Study (MACS), Tasmanian Longitudinal Health Study (TAHS) and the Australian arm of the European Community Respiratory Health Survey (ECRHS).

**Results:**

Among children aged under 6 years, 28.8%–35.6% have ever‐had eczema, and 16.7%–26.6% had ‘current eczema’. Among those aged 6–12 years, 14.6%–24.7% had ‘current eczema’ with 12.0%–18.5% of those at ages of 6 and 10 years classified as having moderate‐to‐severe eczema according to the Scoring of Atopic Dermatitis (SCORAD) index. In adults, the prevalence of ‘eczema ever’ ranged between 13.8% and 48.4%. The 12‐month period prevalence of eczema was 15.1% at age 18, while current eczema was 8.5% at an average age of 51, and 8.8% at an average age 53 years. Eczema was more common among young boys, but this difference became non‐significant for older children and early adolescents. In contrast, eczema was more common for adult women than men.

**Conclusions:**

Eczema is common both in children and adults. The proportion of severe eczema in children was substantial.

## INTRODUCTION

Eczema (atopic dermatitis) is a highly prevalent inflammatory skin disease characterized by skin dryness and itchy lesions.^1^, ^2^ It is strongly associated with other allergic conditions including asthma,^3^ allergic rhinitis and food allergies.^4^

Globally, the 12‐month prevalence rate of eczema was reported to be 13.5%–41.9% in children aged 6‐month up to 18 years,^5^ and ranged between 2.2% and 17.6% in adults.^6^, ^7^ However, eczema is known to have significant geographical variation both within and between countries.^6^, ^8^, ^9^ For instance, in the International Study of Asthma and Allergies in Childhood (ISAAC) Phase Three, the prevalence of ‘current eczema’ ranged from: 0.9% in India to 22.5% in Ecuador for the age group 6–7 years, and 0.2% in China to 24.6% in Columbia among adolescents aged 13–14 years. In Australia, the life‐time prevalence of eczema from birth to the age of 44 years was reported as 41.6%, with females being more commonly affected (48.4%) than males (35.9%).^10^

Childhood eczema is considered to be the starting point for the “atopic march”, which describes the potential progression from atopic dermatitis to asthma and allergic rhinitis over time, usually but not necessarily in that order.^3^, ^10^ Thus, childhood eczema is also a strong predictor of food allergies in children, and significantly associated with subsequent incident asthma throughout adult life.^11^ Approximately, 60% of eczema cases develop in the first year of life, and it can persist to adolescence and adulthood.^10^, ^12^

Eczema is a well‐recognized burden in terms of morbidity, quality of life and healthcare costs.^13^ Furthermore, severe cases of eczema are associated with substantial psychosocial distress and systemic comorbidities.^7^ For instance, children with severe eczema are at risk of challenging behavioural problems and their parents struggle to manage the condition successfully, with more severe disease associated with greater parental stress.^14^ Childhood eczema that persists into adulthood is frequently accompanied by a higher burden of other allergic diseases.

Most previous Australian epidemiological studies on eczema have been carried out in children. However, published data on the epidemiology of eczema in Australian adults were limited,^6^ making it difficult to examine trends in the prevalence of eczema from childhood to adulthood. Therefore, designing studies to better understand the epidemiology of eczema from childhood through adolescence to adulthood is important. However, this has been limited by practical challenges such as the fluctuating nature of eczema^15^, ^16^ and the need for a long duration of follow up, which can be costly and logistically difficult.

Hence, to make the best use of available data, we aimed to present those from already established cohorts in Australia to describe the epidemiology of eczema in the Australian population across the life course, albeit mainly cross‐sectionally for different age epochs. Thus, this study aimed to measure the ***prevalence*** (lifetime, 12‐month period or current prevalence), ***incidence*** (new onset) and ***severity*** of eczema among Australians from childhood to adulthood.

## METHODS

### Data sources

The project was undertaken by obtaining data from four cohort studies: the HealthNuts study,^17^ Melbourne Allergy Cohort Study (MACS),^18^ Tasmanian Longitudinal Health Study (TAHS)^19^ and the Melbourne arm of the European Community Respiratory Health Survey (ECRHS). These data were analysed to describe the prevalence, incidence and severity of eczema among a wide age range of the Australian population (from infancy to middle age). Because the cohorts varied by sampling frame, time periods, underlying risk of atopy and ages, it was not possible to combine the datasets from these studies.

Ethics was approved by Monash University Human Research Ethics Committee (Project Number: 22646), the Research Ethics and Governance office of the Royal Children's Hospital Melbourne (Project Number: 32294) and the Office of Research Ethics and Integrity of the University of Melbourne.

### The HealthNuts study

This study recruited a population‐based sample of infants (*n* = 5276) between 2007–2011 and investigated the natural history and risk factors for developing allergic disorders including food allergy, asthma, hay fever and eczema in children. Participants were recruited at the age of 12 months from council‐run immunization sessions across Melbourne. Parents completed questionnaires and researcher observed eczema assessments were conducted.^17^

In the recruitment questionnaire, data were collected on itchy skin rashes, medication used for rashes and parental‐report of doctor‐diagnosed eczema. At the ages of 4, 6 and 10 years, the entire cohort was approached to complete a questionnaire which included the validated ISAAC questions.^20^ In addition, the duration, age of onset, remission of eczema in the preceding 12 months, frequency of night‐time itch and the use of steroid medications were assessed. Furthermore, at 6 and 10 years of age, an assessment with the Scoring of Atopic Dermatitis severity (SCORAD) was conducted. SCORAD is a widely used instrument to assess the clinical signs of eczema severity with scores ranging between 0 and 103 points, and categorized as mild if the score <25, moderate if 25–50 and severe if >50.^21^, ^22^ The detailed methods and questions used to assess eczema at each follow up are presented in Table 1.

**TABLE 1:** Eczema assessments and definitions by cohort study and age of participants in the four cohort studies

Data source	Age (years)	Eczema assessment (definition)
HealthNuts	1	*Current eczema*: Parent report of a doctor‐diagnosis of eczema
	4, 6 & 10	*Current eczema*: ISAAC criteria^20^ *Doctor‐diagnosed eczema ever*: Parent report *Severity of eczema*: SCORAD index^22^ One or more nights per week of sleep disturbance^8^
MACS	0–2	Doctor‐diagnosed eczema or any rash, treated with topical steroid preparation (excluding rash that only affected the scalp or nappy region)
	3–7	One or more episodes of eczema in 12 months
	12	*Current eczema*: ISAAC^a^ criteria
	18 & 25	history of eczema and either one or more episodes of eczema in 12 months or the use of eczema medications. *Current eczema*: ISAAC^a^ criteria
TAHS	7	*Any eczema*: infantile/flexural/generalized *Clinically assessed eczema*: flexural, generalized
	12	*Current eczema*: flexural, generalized eczema
	18	Eczema since previous survey (after mean age 12 years)
	30	*Eczema ever*: ever had one or more episodes of eczema
	43	*Eczema ever*: ever had eczema or any kind of skin allergy
	53	*Current eczema*: ISAAC criteria
ECRHS^b^	I & II	*Eczema ever*: “Have you ever had eczema or any kind of skin allergy?”
	III	*Eczema ever*: “Have you ever had eczema or any kind of skin allergy?” *Current eczema*: ISAAC criteria

*Note*: *New onset eczema*: If participants did not have eczema in previous surveys and only reported having eczema in the most recent survey, they were classified as 'new onset' eczema, otherwise considered as ‘pre‐existing’ eczema.

^a^ ISAAC definition of ‘ *current eczema’* : a positive response to all three questions: ‘*Have you (your child) ever had an itchy rash that was coming and going for at least 6 months?*’ *AND* ‘*Have you (your child) had this itchy rash in the last 12 months?*’ AND ‘*Has this itchy rash at any time affected any of the following places: the folds of the elbows, behind the knees, in front of the ankles, under the buttocks or around the neck, ears or eyes?*’

^b^ See text for description of age ranges in ECRHS waves.

### Melbourne Atopic Cohort Study (MACS)

MACS was a single centre birth cohort of 620 babies, born between 1990 and 1994, and their families, who were recruited from antenatal clinics in Melbourne.^18^ Only families with a history of allergic disease were eligible. The babies were assessed regularly every 4 weeks from birth to 14 months, then at 18 months and 2 years, annually from ages 3 to 7 years and then at mean ages of 12, 18 and 25 years. Eczema was assessed at each follow up. A summary of methods and questionnaires employed at each follow up is presented in Table 1.

### Tasmanian Longitudinal Health Study (TAHS)

Tasmanian Longitudinal Health Study was commenced in 1968 when the parents of all Tasmanian schoolchildren (probands) born in 1961 (who were aged 7 years at the time of the baseline survey) were recruited. Parents completed questionnaires for 8583 probands (99%) and 21,043 siblings, as well as for themselves. Subsequent follow‐up studies were conducted when the participants were aged 12, 18, 30, 43 and 53 years.^19^ Eczema assessment questions are summarized in Table 1.

### European Community respiratory health survey (ECRHS Melbourne site)

The ECRHS study was developed in response to a perceived increase in the prevalence of asthma and allergic diseases. For the first wave of the cohort conducted in 1990–1994 (ECRHSI), young adults aged between 20 and 44 years (*n* = 876 in the Australian centre in Melbourne) were selected at random from population‐based registers. Follow‐ups were carried out from 1998 to 2002 (ECRHSII) and 2008 to 2014 (ECRHSIII).

Eczema was assessed in all the three waves. Unfortunately, the questions used were not consistent. In ECRHS‐I and II, only life‐time prevalence of eczema was assessed (see Table 1). During the ECHRS III, current prevalence of eczema was assessed using the ISAAC questionnaire.

## RESULTS

### Baseline characteristics of participants

The baseline characteristics of participants included in HealthNuts, MACS, TAHS and ECRHS are presented in Table 2.

**TABLE 2:** Baseline characteristics of participants in the four cohorts, *n* (%)

Characteristics	HealthNuts (*n* = 4954)	MACS (*n* = 619)	TAHS (*n =* 8583)	ECRHS‐I (*n* = 876)
Male	2521 (50.9)	316 (51.1)	4393 (51.2)	413 (47.2)
Average age at recruitment	1 year	At birth	6.5 ± 0.3 years	34.7 ± 6.8 years
Number of siblings				
0	2479 (48.5)	66 (16.5)	923 (10.8)	54 (6.2)
1	1734 (33.9)	191 (47.8)	1742 (20.3)	238 (27.2)
2	668 (13.1)	100 (25.0)	2392 (27.9)	250 (28.5)
3	178 (3.5)	33 (8.2)	1695 (19.8)	149 (17.0)
4+	57 (1.1)	10 (2.5)	1882 (21.3)	185 (21.1)
Parental smoking				
Mother smoked ever	1697 (33.3)	155 (25.1)	3033 (37.7)	289 (33.0)
Father smoked ever	1958 (39.7)	203 (33.2)	4, 819 (61.5)	526 (60.1)
Allergic conditions in siblings				
Sibling with asthma	–	219 (35.3)	2265 (10.7)	–
Sibling with hay fever	–	135 (21.8)	1263 (6.1)	–
Sibling with eczema	–	237 (48.7)	1950 (9.3)	–
Allergic conditions in parents				
Maternal asthma	–	268 (43.3)	883 (11.0)	114 (13.2)
Maternal hay fever	–	375 (60.6)	1830 (22.8)	–
Maternal eczema	–	241 (38.9)	–	311 (35.5)
Maternal food allergy	–	239 (38.6)	–	–
Paternal asthma	–	158 (25.7)	853 (10.8)	84 (9.6)
Paternal hay fever	–	284 (46.2)	1353 (17.3)	–
Paternal eczema	–	126 (20.5)	–	224 (25.6)
Paternal food allergy	–	131 (21.3)	–	–
Allergic conditions in any immediate family^a^ member				
Allergic rhinitis	2843 (53.9)	–	–	–
Asthma	3619 (30.7)	–	–	–
Dermatitis	765 (14.5)	–	–	–
Eczema	1611 (30.5)	–	–	–
Hay fever	2640 (50.0)	–	–	–
Food allergy	577 (11.2)	–	–	–

^a^ *Immediate family*: mother, father or sibling.

From a total of 5276 participants in HealthNuts, 4954 children (50.9% males) had complete data on detailed eczema questions at age of 1 year and were included in this analysis. Of those, 3030 (61.2%), 3267 (65.9%) and 2958 (59.7%) also had full questionnaire assessments that included eczema using the ISAAC questionnaires at ages of 4, 6 and 10 years. Those lost to follow up were not significantly different by sex compared with those who continued in the study.

In the MACS cohort, a total of 619 probands (51.1% males) were assessed for eczema at baseline. Because of the inclusion criteria, the prevalence of allergic conditions in family members was high.

A total of 8583 children (51.2% males) were recruited in the TAHS baseline survey in 1968, then aged between 5.1 and 7.9 years (mean ± SD, 6.5 ± 0.3 years) and over half (51.2%) were male. Overall, 96.3% of the children were born in Australia.

The Australian baseline ECRHS included a sample of 876 young adults, who were then aged between 20 and 44 years (mean ± SD, 34.7 ± 6.8 years), 80.3% were born in Australia and over half (52.9%) were female. Overall, 72.7% of participants who took part in ECRHSI also took part in ECRHSII, and 36.3% in ECRHSIII; responders were older and more likely to be women than non‐responders. However, there was no significant difference in the prevalence of reported ‘eczema or skin allergy’ at baseline (ECRHSI) between those who did or did not take part in ECRHSII or ECRHSIII surveys.

### Prevalence of eczema in children

**FIGURE 1:**
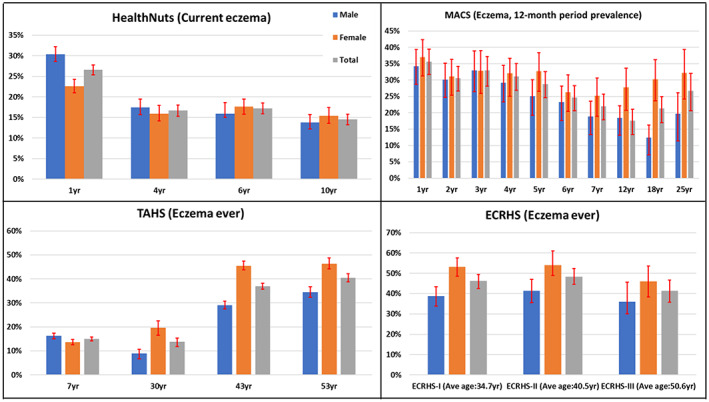
Prevalence (95% CI as red lines) of eczema by age and sex in the four cohort studies.

**FIGURE 2:**
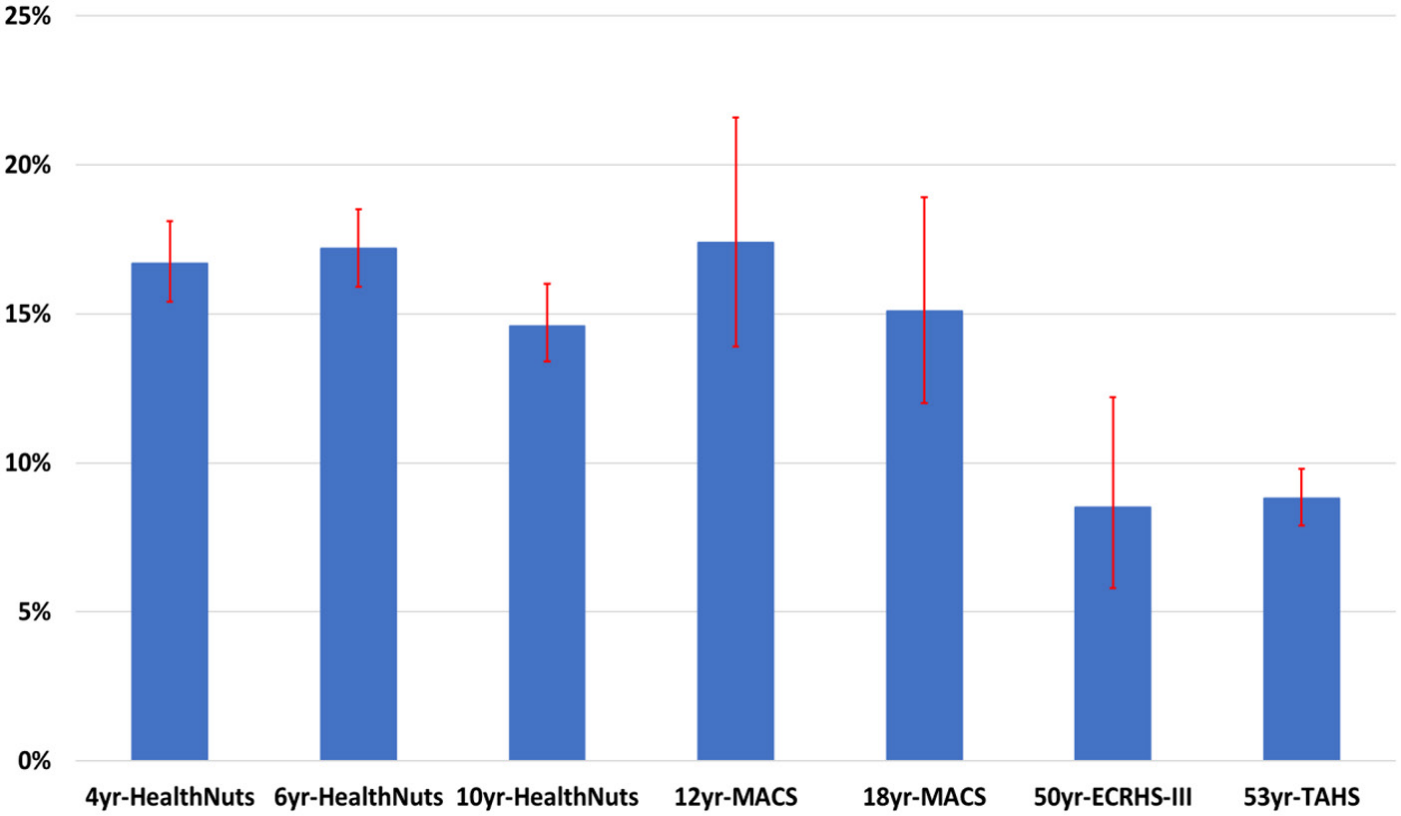
Prevalence (95% CI as red lines) of ‘current eczema’ in children and adults according to ISAAC criteria.

In HealthNuts, at an average age of 1‐year, the prevalence of parent‐reported eczema was 26.6% (95% CI: 25.4%–27.8%) (Figure 1). According to ISAAC criteria, around one in every six children at ages of 4, 6 or 10 years had current eczema (Figure 2).

In MACS, 46.1% (95% CI: 42.0%–50.2%) of children under 2 years of age ever had eczema. The prevalence of eczema in those aged 0–1 years was 35.6% (31.8%–39.5%), and for 1–2 years it was 30.6% (27.0%–34.5%). For annual follow ups between ages of 3 and 7 years the prevalence of eczema declined from 36.6% to 18.3% (Figure 1). By ISAAC criteria, the prevalence of current eczema was 17.6% (14.1%–21.9%) at an average age of 12 years (Figure 2).

During the baseline TAHS survey (at average age 7 years), the prevalence of infantile, flexural and generalized eczema was 9.9% (95% CI: 9.3%–10.5%), 9.7% (9.1%–10.3%) and 1.0% (0.8%–1.3%), respectively. The prevalence of clinically assessed flexural eczema at one time point was 2.3% (2.0%–2.6%), and 15.0% (14.2%–15.8%) had ‘any eczema’. Due to sampling and questionnaire differences, it was not possible to describe the prevalence and incidence of eczema for the follow up surveys in 1974 and 1981. However, the prevalence of flexural and generalized eczema was 2.9% and 1.7% in a sub‐sample of the TAHS study at age of 12 years.

### Prevalence of eczema in adults

The number of participants decreased over follow up of the MACS cohort over time (*n* = 424 at 18, and *n* = 273 at 25 years). Prevalence of eczema at age of 2 years was not associated with completion of the 18‐ and 25‐year follow‐ups. The 12‐month period‐prevalence of eczema in participants aged 18 and 25 years was 21.3% (95% CI: 17.7%–25.6%) and 26.7% (21.7%–32.4%) respectively.

Among adult participants of TAHS, the lifetime prevalence of eczema was 13.8% (12.2%–15.7%), 37.0% (35.8%–38.3%) and 40.5% (38.9%–42.2%) at ages of 30, 43 and 53 years, respectively (Figure 1).

Among ECRHS participants, the lifetime prevalence of eczema was 46.3% (43.1%–50.0%) for ECRHSI, 48.4% (44.5–52.2%) for ECRHSII and 41.4% (36.1–47.0%) for ECRHSIII (Figure 1). When adjusted for age and gender, the prevalence of ‘eczema ever’ during ECRHSII was 31.1% (28.5–33.8%) and ECRHSIII 27.0% (23.1–31.0%).

### Eczema by age and sex of participants

The descriptive epidemiology of eczema by age and sex of participants in the four cohorts is presented in Figure 1. In HealthNuts, the prevalence of eczema in infants was significantly higher among boys than girls (30.4 vs 22.6%, *p* < 0.001); however, there was no significant difference by sex at later ages. In MACS, girls had a higher prevalence of eczema than did boys; however, this difference only become statistically significant during adolescence (*χ* ^2^ = 4.8, *p* < 0.05 at 12 years). Among adults, females were more likely to have eczema than males (OR = 3.06; 95% CI: 1.84–5.07 at age of 18 years, and OR = 1.94; 1.10–3.44 at age 25 years).

In TAHS, at age of 7 years both infantile and flexural eczema were more common among boys than girls (*p* < 0.01). However, by 30, 43‐ and 53‐years, females were more likely to have eczema than males. Similarly, in the ECRHS cohort ‘eczema ever’ was significantly higher for females than males (ECRHSI *p* < 0.001; ECRHSII *p* < 0.01; and *p* = 0.071 in ECRHSIII).

Summarized findings from each cohort study by age groups, the prevalence of ‘eczema ever’, 12‐month period or ‘current eczema’, as well as some severity measures, are presented in Table S1.

Since the ISAAC questionnaire was used by all four cohorts at some survey point(s), we calculated the current prevalence of eczema at ages of 4, 6 and 10 years (HealthNuts), 12 and 18 years (MACS), at average age of 50.6 years (ECRHSIII) and 53 years (TAHS). As shown in Figure 2, the prevalence of current eczema according to the ISAAC criteria was 13.4%–21.6% in children and 5.8%–18.9% in adults.

### Body areas affected by eczema

Eczema most commonly affected the folds of the elbows (62.1%), behind the knees (56.8%), and the buttocks (33.3%) in children aged 12 in MACS. In TAHS at age 53 years, around the neck, eyes and ears (29.8%), folds of the elbows (29.7%) and behind the knees (27.7%) were the commonly affected areas in adults.

### Age of onset of eczema

**FIGURE 3:**
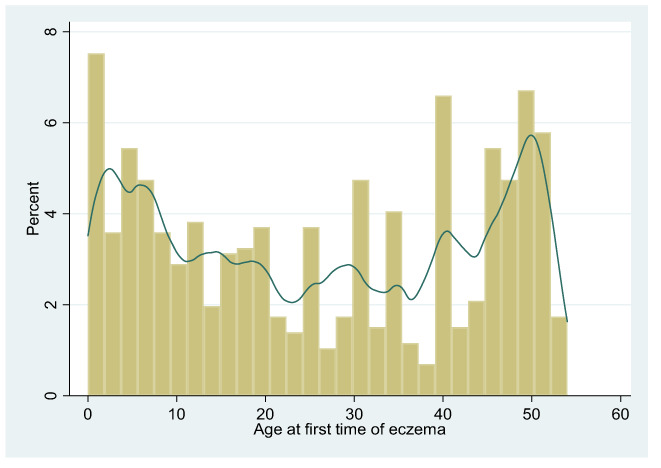
Age at first time of eczema as reported by TAHS participants at age of 53 years follow up.

In the HealthNuts baseline survey, over three‐quarters (78.5%) of infants were reported to have developed it by the age of 6‐month. However, among adult participants of TAHS surveyed at age 53 years, the reported median age at onset of eczema was 26 years (interquartile range 10–44), and two‐thirds (68.8%) reported first having had eczema as adults. In contrast, the distribution of self‐reported age of first eczema presentation among adult participants of the TAHS followed a bimodal distribution in which new onset eczema peaked during infancy and around middle age (Figure 3).

### New onset eczema

**FIGURE 4:**
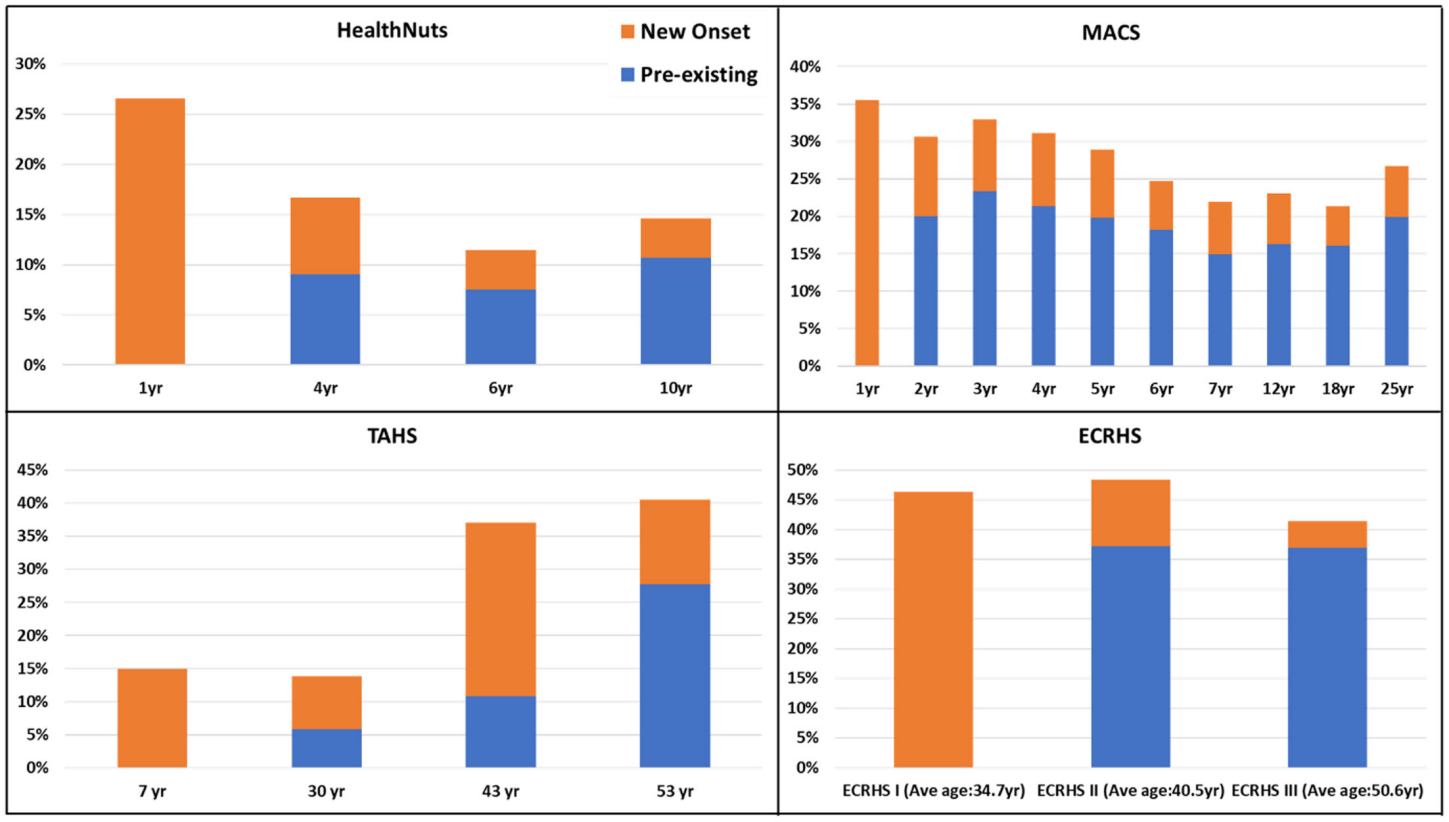
Pre‐existing and new onset eczema by age of participants in each cohort study (*new onset*: If participants had current eczema for the first time during the most recent survey. For instance, new onset eczema at age of 25 years was if the participant had ‘current eczema’ at 25 but never in any of the previous surveys).

Among children and adults with eczema investigated, the majority had pre‐existing disease (Figure 4). The proportion of new onset eczema followed a decreasing trend with age. During the second wave of the ECRHS, nearly a quarter (23.1%) of those who ever had eczema or any itchy skin lesion did not have the condition during the first wave. Similarly, a further 10.8% of those who had eczema during the ECRHSIII had it for the first time, i.e. did not have the condition during previous surveys.

### Eczema severity (HealthNuts)

According to the SCORAD classification, 12.1% (9.1%–15.8%) of current eczema at age 6 years and 18.5% (15.3%–22.0%) at age 10 years were classified as moderate to severe. At 4, 6 or 10 years, 7.0%–7.9% of the children with ‘current eczema’ were kept awake at least one night per week due to their itchy rash, and 10.7%–12.3% had been treated with topical steroids for over 10 days in a row.

## DISCUSSION

By analysing data from four cohort studies among a wide range of age groups, including children, adolescents and adults, we have described the epidemiology of eczema from infancy to the 6th decade of life in south eastern Australia (Melbourne, Victoria and Tasmania). The prevalence of ‘*eczema ever*’, 12‐month period‐prevalence and ‘*current eczema*’ were high in Australian children and adults.

Almost one third of children aged under 6 years of age ever had eczema, while nearly a quarter had ‘*current eczema*’. This was similar to previous Western Australian report of at least 1 in 3 children under 5 years of age having eczema.^23^ Among children aged 6–12 years, nearly 1 in every 5 children had ‘*current eczema*’, which was also similar to previous reports from two population‐based birth cohorts from the UK.^24^ In MACS, a cohort of children with a family history of allergic disease, almost one in every five young adults (aged 18 and 25 years) had ‘current eczema’, while among middle‐aged adults in TAHS the prevalence of ‘eczema ever’ was also high. Almost a third of TAHS participants at ages 43 and 53 years, and close to half of the ECRHS‐II & III participants at average ages 40.5 and 50.6 years ever had eczema, which was comparable to previous Australian and international reports.^10^, ^25^

These high rates of eczema are consistent with international findings. In the current analysis, we described the prevalence of ‘current eczema’ in both children and adults using ISAAC criteria, with 13.4%–21.6% of children having ‘current eczema’, according to this definition. This is consistent with a recently conducted international survey of children and adolescents (6 months to <18 years old) from 18 countries that reported a 13.5%–41.9% prevalence of eczema according to these same criteria.^5^ For example, the prevalence of eczema was 16.5% in the US, 25.4% in Canada, 24.0% in the UK,^5^ and 17.1% in Australia.^8^ In the current analysis, the prevalence of eczema was higher among MACS participants, but the MACS study was a high‐risk cohort in that all infants recruited came from families with a history of allergic diseases,^18^ so this estimate may well not be fully representative of the general population.

Eczema was more common among boys at early ages, but this difference became non‐significant in older children and early adolescents. Eczema was more common in female than male adults. This is consistent with previous reports of a lower prevalence of eczema in male adults and older age groups,^7^, ^9^ and a lack of association of eczema with sex in children and adolescents.^5^ Adolescence is known to be an important period of potential change in the body's response to allergens when eczema decreases in prevalence.^26^

Age at first‐presentation of eczema in the TAHS cohort, as assessed at age of 53 years using the ISAAC questionnaire, seemed to follow a bimodal distribution in which new onset eczema peaked during infancy and around middle age. This bimodal distribution of eczema across the lifespan could potentially be limited by recall bias, with participants questioned during their sixth decade of life. Previous studies have demonstrated that remembering having eczema in childhood was mainly possible if it was of long duration in childhood or adult onset type.^27^ Adults might not accurately recall whether they had mild allergic conditions as children.^28^

At ages of 6 and 10 years in the HealthNuts study, 12.0% and 18.5% of children respectively with ‘current eczema’ were found to have moderate–severe disease according to the SCORAD. In the ISAAC Phase‐3 global study, severe eczema was defined as ‘current eczema’ associated with one or more nights per week of sleep disturbance.^8^ In HealthNuts, about 10% of children with ‘current eczema’ fulfilled this criterion; furthermore, at least 10% of children with eczema were treated with topical steroids for over 10 days in a row. This use of steroids could be reasonably considered as a measure of severity of eczema, as previous studies also reported more frequent use of such preparations by children with moderate–severe eczema than by children with mild eczema.^29^ Combining these proxy measures of eczema severity, in the HealthNuts cohort about 10%–20% children with eczema had a more severe form. This is substantial and could have major impacts on the quality of life of children and families, since severe eczema is known to be associated with impaired sleep, poorer health outcomes and increased healthcare utilization.^30^ A recently conducted international survey of children and adolescents from 18 countries reported that, among children with eczema, the prevalence of severe eczema was usually less than 15%, although this varied across age groups and countries.^5^

Our study has multiple strengths. Firstly, it was based on multiple cohorts that collected data from a diverse age range of participants (infants, children, adolescents and adults). We were able to describe the prevalence of eczema using the internationally accepted ISAAC definition from all cohorts which enabled a consistency of evaluation for ‘current eczema’ across age groups. The ISAAC questions are known to provide adequate prevalence estimates at the population level.^20^ In some cohorts, flexural eczema was also assessed clinically. All our cohorts were from mainly populations of European heritage, which limited generalizability, in the current multi‐ethnic population of Australia. However, it does provide a strong benchmark for future multi‐ethnic comparisons. The cohorts were recruited between 1968 and 2011, providing a uniquely long perspective; however, the obverse is that period effects could well have affected the results.

It should be noted that comparisons of eczema prevalence could be affected by several limitations. Eczema assessment questions varied considerably between cohorts and at different waves of each cohort. This made it difficult to compare results either between or within cohorts. Another limitation is that eczema severity measures were lacking in three of the four cohorts and hence we were not able to report on severity of eczema among adults. Finally, the inclusion of MACS was problematic in that participants came from high risk families, rather than the general population. Indeed, the numerical signals for eczema were as expected somewhat higher, but at least it gave some comparative perspective to the range of eczema epidemiology data in Australia.

In conclusion, eczema is common in both Australian children and adults. It is more common among boys in childhood, and among women in adulthood. It is also higher among participants with a positive family history of eczema. A more definitive estimate of the epidemiology and risk factors for eczema in Australia could be determined by designing a longitudinal national cohort study primarily aimed at eczema assessment and using consistent validated methods across the life span and in a more diverse sample of the general population. This summary of the epidemiology of eczema should ultimately help allocation of resources both by government and the pharmaceutical industry to improve patient outcomes.

## FUNDING INFORMATION

HealthNuts: This work was supported by funding from the National Health and Medical Research Council (NHMRC) of Australia, the Ilhan Food Allergy Foundation, AnaphylaxiStop, the Charles and Sylvia Viertel Medical Research Foundation and the Victorian Government‘s Operational Infrastructure Support Programme. MACS: The first 6 years of the MACS was funded (study formula and staff) by Nestec Ltd, a subsidiary of Nestlé Australia. The 12‐year follow up was funded by a project grant from the Asthma Foundation of Victoria. The NHMRC funded the 18 and 25‐year follow‐up studies. The TAHS was funded by NHMRC; European Union Horizon 2020; the University of Melbourne; Clifford Craig Medical Research Trust of Tasmania; the Victorian, Queensland & Tasmanian Asthma Foundations; the Royal Hobart Hospital Research Foundation; Helen MacPherson Smith Trust; GlaxoSmithKline and Lions Club of Penguin. ECRHS was conducted in Australia with the support of the Asthma Foundation of Victoria, Allen+Hanburys and NHMRC. All bodies that have funded aspects of any of the cohorts have had no role in interpretation or publication of study findings.

## Supporting information


Table S1.

## References

[1] Asher MI , Montefort S , Bjorksten B , Lai CK , Strachan DP , Weiland SK , et al. Worldwide time trends in the prevalence of symptoms of asthma, allergic rhinoconjunctivitis, and eczema in childhood: ISAAC phases one and three repeat multicountry cross‐sectional surveys. Lancet. 2006;368(9537):733–43. 16935684 10.1016/S0140-6736(06)69283-0

[2] Johansson SG , Bieber T , Dahl R , Friedmann PS , Lanier BQ , Lockey RF , et al. Revised nomenclature for allergy for global use: report of the nomenclature review Committee of the World Allergy Organization, October 2003. J Allergy Clin Immunol. 2004;113(5):832–6. 15131563 10.1016/j.jaci.2003.12.591

[3] Dharmage SC , Lowe AJ , Matheson MC , Burgess JA , Allen KJ , Abramson MJ . Atopic dermatitis and the atopic march revisited. Allergy. 2014;69(1):17–27. 24117677 10.1111/all.12268

[4] Martin PE , Eckert JK , Koplin JJ , Lowe AJ , Gurrin LC , Dharmage SC , et al. Which infants with eczema are at risk of food allergy? Results from a population‐based cohort. Clin Exp Allergy. 2015;45(1):255–64. 25210971 10.1111/cea.12406

[5] Silverberg JI , Barbarot S , Gadkari A , Simpson EL , Weidinger S , Mina‐Osorio P , et al. Atopic dermatitis in the pediatric population: a cross‐sectional, international epidemiologic study. Ann Allergy Asthma Immunol. 2021;126(4):417–28 e2. 33421555 10.1016/j.anai.2020.12.020

[6] Harrop J , Chinn S , Verlato G , Olivieri M , Norback D , Wjst M , et al. Eczema, atopy and allergen exposure in adults: a population‐based study. Clin Exp Allergy. 2007;37(4):526–35. 17430349 10.1111/j.1365-2222.2007.02679.x

[7] Silverberg JI , Hanifin JM . Adult eczema prevalence and associations with asthma and other health and demographic factors: a US population‐based study. J Allergy Clin Immunol. 2013;132(5):1132–8. 24094544 10.1016/j.jaci.2013.08.031

[8] Odhiambo JA , Williams HC , Clayton TO , Robertson CF , Asher MI , ISAAC Phase Three Study Group . Global variations in prevalence of eczema symptoms in children from ISAAC phase three. J Allergy Clin Immunol. 2009;124(6):1251–8 e23. 20004783 10.1016/j.jaci.2009.10.009

[9] Barbarot S , Auziere S , Gadkari A , Girolomoni G , Puig L , Simpson EL , et al. Epidemiology of atopic dermatitis in adults: results from an international survey. Allergy. 2018;73(6):1284–93. 29319189 10.1111/all.13401

[10] Burgess JA , Dharmage SC , Byrnes GB , Matheson MC , Gurrin LC , Wharton CL , et al. Childhood eczema and asthma incidence and persistence: a cohort study from childhood to middle age. J Allergy Clin Immunol. 2008;122(2):280–5. 18572229 10.1016/j.jaci.2008.05.018

[11] Martin PE , Koplin JJ , Eckert JK , Lowe AJ , Ponsonby AL , Osborne NJ , et al. The prevalence and socio‐demographic risk factors of clinical eczema in infancy: a population‐based observational study. Clin Exp Allergy. 2013;43(6):642–51. 23711126 10.1111/cea.12092

[12] Mortz CG , Andersen KE , Dellgren C , Barington T , Bindslev‐Jensen C . Atopic dermatitis from adolescence to adulthood in the TOACS cohort: prevalence, persistence and comorbidities. Allergy. 2015;70(7):836–45. 25832131 10.1111/all.12619

[13] Jenner N , Campbell J , Marks R . Morbidity and cost of atopic eczema in Australia. Australas J Dermatol. 2004;45(1):16–22. 14961903 10.1111/j.1440-0960.2004.00046.x

[14] Mitchell AE , Fraser JA , Ramsbotham J , Morawska A , Yates P . Childhood atopic dermatitis: a cross‐sectional study of relationships between child and parent factors, atopic dermatitis management, and disease severity. Int J Nurs Stud. 2015;52(1):216–28. 25441758 10.1016/j.ijnurstu.2014.09.008

[15] Johansson EK , Ballardini N , Bergstrom A , Kull I , Wahlgren CF . Atopic and nonatopic eczema in adolescence: is there a difference? Br J Dermatol. 2015;173(4):962–8. 25970379 10.1111/bjd.13901

[16] Kramer U , Weidinger S , Darsow U , Mohrenschlager M , Ring J , Behrendt H . Seasonality in symptom severity influenced by temperature or grass pollen: results of a panel study in children with eczema. J Invest Dermatol. 2005;124(3):514–23. 15737191 10.1111/j.0022-202X.2005.23625.x

[17] Koplin JJ , Wake M , Dharmage SC , Matheson M , Tang ML , Gurrin LC , et al. Cohort profile: the HealthNuts study: population prevalence and environmental/genetic predictors of food allergy. Int J Epidemiol. 2015;44(4):1161–71. 25613427 10.1093/ije/dyu261

[18] Lowe AJ , Lodge CJ , Allen KJ , Abramson MJ , Matheson MC , Thomas PS , et al. Cohort profile: Melbourne Atopy Cohort Study (MACS). Int J Epidemiol. 2017;46(1):25–6. 27097746 10.1093/ije/dyw011

[19] Matheson MC , Abramson MJ , Allen K , Benke G , Burgess JA , Dowty JG , et al. Cohort profile: the Tasmanian Longitudinal Health STUDY (TAHS). Int J Epidemiol. 2017;46(2):407–8i. 27272183 10.1093/ije/dyw028

[20] Asher MI , Keil U , Anderson HR , Beasley R , Crane J , Martinez F , et al. International study of asthma and allergies in childhood (ISAAC): rationale and methods. Eur Respir J. 1995;8(3):483–91. 7789502 10.1183/09031936.95.08030483

[21] Rehal B , Armstrong AW . Health outcome measures in atopic dermatitis: a systematic review of trends in disease severity and quality‐of‐life instruments 1985‐2010. PLoS One. 2011;6(4):e17520. 21533286 10.1371/journal.pone.0017520 PMC3076368

[22] Schmitt J , Langan S , Deckert S , Svensson A , von Kobyletzki L , Thomas K , et al. Assessment of clinical signs of atopic dermatitis: a systematic review and recommendation. J Allergy Clin Immunol. 2013;132(6):1337–47. 24035157 10.1016/j.jaci.2013.07.008

[23] Owens L , Laing IA , Zhang G , Turner S , Le Souef PN . Prevalence of allergic sensitization, hay fever, eczema, and asthma in a longitudinal birth cohort. J Asthma Allergy. 2018;11:173–80. 30147342 10.2147/JAA.S170285 PMC6095121

[24] Belgrave DC , Granell R , Simpson A , Guiver J , Bishop C , Buchan I , et al. Developmental profiles of eczema, wheeze, and rhinitis: two population‐based birth cohort studies. PLoS Med. 2014;11(10):e1001748. 25335105 10.1371/journal.pmed.1001748 PMC4204810

[25] Ronmark EP , Ekerljung L , Lotvall J , Wennergren G , Ronmark E , Toren K , et al. Eczema among adults: prevalence, risk factors and relation to airway diseases. Results from a large‐scale population survey in Sweden. Br J Dermatol. 2012;166(6):1301–8. 22372948 10.1111/j.1365-2133.2012.10904.x

[26] Tai A , Tran H , Roberts M , Clarke N , Wilson J , Robertson CF . Trends in eczema, rhinitis, and rye grass sensitization in a longitudinal asthma cohort. Ann Allergy Asthma Immunol. 2014;112(5):437–40. 24767696 10.1016/j.anai.2014.03.005

[27] Mortz CG , Andersen KE , Bindslev‐Jensen C . Recall bias in childhood atopic diseases among adults in the Odense Adolescence Cohort Study. Acta Derm Venereol. 2015;95(8):968–72. 25916800 10.2340/00015555-2128

[28] Burgess JA , Walters EH , Byrnes GB , Wharton C , Jenkins MA , Abramson MJ , et al. Who remembers whether they had asthma as children? J Asthma. 2006;43(10):727–30. 17169822 10.1080/02770900601028587

[29] Ballardini N , Kull I , Soderhall C , Lilja G , Wickman M , Wahlgren CF . Eczema severity in preadolescent children and its relation to sex, filaggrin mutations, asthma, rhinitis, aggravating factors and topical treatment: a report from the BAMSE birth cohort. Br J Dermatol. 2013;168(3):588–94. 23445315 10.1111/bjd.12196

[30] Silverberg JI , Simpson EL . Association between severe eczema in children and multiple comorbid conditions and increased healthcare utilization. Pediatr Allergy Immunol. 2013;24(5):476–86. 23773154 10.1111/pai.12095 PMC4397968

