# Supplementary material for: Epidemiology of eczema in South‐Eastern Australia
Source: Australas J Dermatol. 2022 Dec 19;64(1):e41–50. doi: 10.1111/ajd.13966 (PMC10952653; doi:10.1111/ajd.13966)
Supplement: Supplementary file 1 — Table S1. [file AJD-64-e41-s001.docx]

| **Supplementary table S1: Epidemiology of eczema from four Australian cohorts by age group of participants** | | | | | | | |
| --- | --- | --- | --- | --- | --- | --- | --- |
| **Age group (years)** | **Data source** | **Average age (years)** | **N** | **Prevalence, % (95%CI)** | **Eczema cleared in 12 months (%)** | **Severity of eczema (%)** | **Description/definition** |
| **0-5** | **MACS** | 1 | 619 | 35.6 (31.8-39.5) | - | - | ≥ 1 Episodes of eczema in 12 months |
|  |  | 2 | 575 | 30.6 (27.0-34.5) | - | - |  |
|  |  | 3 | 419 | 32.9 (27.6-36.6) | - | - |  |
|  |  | 4 | 508 | 31.1 (27.2-35.3) | - | - |  |
|  |  | 5 | 496 | 28.8 (25.0-27.8) | - | - |  |
|  | **HealthNuts** | 1 | 4,954 | 26.6 (25.4-27.8) | - | - | Itchy rash, eczema or dry skin, other than nappy area |
|  |  | 4 | 3,030 | 16.7 (15.4-18.1) | 70.3 |  | *Current Eczema*: ISAAC Criteria |
| **6-12** | **MACS** | 6 | 489 | 24.7 (21.1-28.8) | - | - | ≥ 1 Episodes of eczema in 12 months |
|  |  | 7 | 442 | 21.9 (18.3-26.1) | - | - |  |
|  |  | 12 | 369 | 17.6 (13.9-21.9) | - | - | *Current Eczema*: ISAAC Criteria |
|  | **HealthNuts** | 6 | 3,267 | 17.2 (15.9-18.5) | 72.3 | 12.1 | *Current Eczema*: ISAAC Criteria, and  *Severity of eczema*: using SCORAD index |
|  |  |  |  |  |  | 8.6 |  |
|  |  | 10 | 2,958 | 14.6 (13.4-16.0) | 78.5 | 18.5 |  |
|  | **TAHS** | 7 | 8,583 | 15.0 (14.2-15.8) |  | - | Any eczema (infantile/flexural/generalised) |
| **18+** | **MACS** | 18 | 417 | 21.3 (17.7-25.6) | - | - | ≥ 1 Episodes of eczema in 12 months |
|  |  | 25 | 216 | 26.7 (21.7-32.4) |  |  | ≥ 1 Episodes of eczema in 12 months |
|  | **TAHS** | 18 | 658 | 23.0 (12.2-15.7) | - | - | Eczema ever |
|  |  | 30 | 1,501 | 13.8 (12.2-15.7 | - | - | Eczema ever |
|  |  | 43 | 5,729 | 37.0 (35.8-38.3) | - | - | Eczema ever |
|  |  | 53 | 5,672 | 40.5 (38.9-42.2) | - | - | Eczema ever |
|  |  |  |  | 8.8 (7.9-9.8) | - | - | *Current Eczema*: ISAAC Criteria |
|  | **ECRHSI** | 34.7 | 876 | 46.3 (43.1-50.0) | - | - | Eczema ever |
|  | **ECRHSII** | 40.5 | 637 | 48.4 (44.5-52.2) | - | - | Eczema ever |
|  | **ECRHSIII** | 50.6 | 318 | 41.4 (36.1-47.0) |  | - | Eczema ever |
|  |  |  |  | 8.5 (5.8-12.2) | - | - | *Current Eczema*: ISAAC Criteria |
